# Mitigating antimicrobial resistance (AMR) using implementation research: a development funder’s approach

**DOI:** 10.1093/jacamr/dlad031

**Published:** 2023-03-27

**Authors:** Mark P Khurana, Sabiha Essack, Ghada Zoubiane, Nandini Sreenivasan, Gloria Cristina Cordoba, Erica Westwood, Anders Dalsgaard, Robinson H Mdegela, Mirfin Mpundu, Rodrigo Scotini, Augustine B Matondo, Alexanda Mzula, Nina Chanishvili, Dimitri Gogebashvili, Maia Beruashvili, Marika Tsereteli, Talant Sooronbaev, Jesper Kjærgaard, Joakim Bloch, Elvira Isaeva, Geoffrey Mainda, Geoffrey Muuka, Ntombi B Mudenda, Fusya Y Goma, Duc-Huy Chu, Duncan Chanda, Uchizi Chirwa, Kaunda Yamba, Kenneth Kapolowe, Sombo Fwoloshi, Lawrence Mwenge, Robert Skov

**Affiliations:** ICARS, International Centre for Antimicrobial Resistance Solutions, Ørestads Boulevard 5, Copenhagen 2300, Denmark; Section of Epidemiology, Department of Public Health, University of Copenhagen, Copenhagen, Denmark; ICARS, International Centre for Antimicrobial Resistance Solutions, Ørestads Boulevard 5, Copenhagen 2300, Denmark; Antimicrobial Research Unit, College of Health Sciences, University of KwaZulu-Natal, Durban, South Africa; ICARS, International Centre for Antimicrobial Resistance Solutions, Ørestads Boulevard 5, Copenhagen 2300, Denmark; ICARS, International Centre for Antimicrobial Resistance Solutions, Ørestads Boulevard 5, Copenhagen 2300, Denmark; ICARS, International Centre for Antimicrobial Resistance Solutions, Ørestads Boulevard 5, Copenhagen 2300, Denmark; ICARS, International Centre for Antimicrobial Resistance Solutions, Ørestads Boulevard 5, Copenhagen 2300, Denmark; ICARS, International Centre for Antimicrobial Resistance Solutions, Ørestads Boulevard 5, Copenhagen 2300, Denmark; Department of Veterinary and Animal Sciences, Faculty of Health and Medical Sciences, University of Copenhagen, Frederiksberg C, Denmark; Department of Veterinary Medicine and Public Health, Sokoine University of Agriculture, Morogoro, Tanzania; ICARS, International Centre for Antimicrobial Resistance Solutions, Ørestads Boulevard 5, Copenhagen 2300, Denmark; ReAct Africa, Lusaka, Zambia; World Diabetes Foundation, Bagsværd 2880, Denmark; Department of Veterinary Medicine and Public Health, Sokoine University of Agriculture, Morogoro, Tanzania; Department of Veterinary Medicine and Public Health, Sokoine University of Agriculture, Morogoro, Tanzania; George Eliava Institute of Bacteriophage Microbiology and Virology, Gotua Street 3, Tbilisi 0160, Georgia; LTD Invet Group, 84a, Vakhushti Bagrationi Street, Tbilisi 0154, Georgia; Ministry of Environmental Protection and Agriculture of Georgia, Marshal Gelovani 6, Tbilisi 0159, Georgia; The Faculty of Veterinary Medicine, European University, Tbilisi, Georgia; Department of Communicable Diseases, National Center for Disease Control and Public Health, Kakheti Highway 99, Tbilisi 0198, Georgia; National Center of Cardiology and Internal Medicine named after academician M. Mirrakhimov, Togolok Moldo Str, 3, Bishkek 720040, Kyrgyzstan; Department of Children and Adolescents, Copenhagen University Hospital, Rigshospitalet, Blegdamsvej 9, Copenhagen 2100, Denmark; Department of Children and Adolescents, Copenhagen University Hospital, Rigshospitalet, Blegdamsvej 9, Copenhagen 2100, Denmark; National Center of Maternity and Childhood Care, Akhunbaev Str, 190, Bishkek 720038, Kyrgyzstan; Department of Public Health, The Research Unit for General Practice and Section of General Practice, University of Copenhagen, Øster Farimagsgade 5, Copenhagen 1354, Denmark; Department of Veterinary Services, Ministry of Fisheries and Livestock, PO Box 50060, Lusaka, Zambia; Department of Veterinary Services, Ministry of Fisheries and Livestock, PO Box 50060, Lusaka, Zambia; School of Veterinary Medicine, University of Zambia, PO Box 32379, Lusaka, Zambia; Department of Veterinary Services, Ministry of Fisheries and Livestock, PO Box 50060, Lusaka, Zambia; Department of Animal Health, Ministry of Agriculture and Rural Development, Ha Noi 115-19, Viet Nam; University Teaching Hospital, Box 17, UTH Post Office, Nationalist Rd., Lusaka, Zambia; Ministry of Health, Ndeke House, Haile Selassie Avenue, PO Box 30205, Lusaka, Zambia; University Teaching Hospital, Box 17, UTH Post Office, Nationalist Rd., Lusaka, Zambia; Ministry of Health, Ndeke House, Haile Selassie Avenue, PO Box 30205, Lusaka, Zambia; School of Veterinary Medicine, University of Zambia, PO Box 32379, Lusaka, Zambia; University Teaching Hospital, Box 17, UTH Post Office, Nationalist Rd., Lusaka, Zambia; University Teaching Hospital, Box 17, UTH Post Office, Nationalist Rd., Lusaka, Zambia; University Teaching Hospital, Box 17, UTH Post Office, Nationalist Rd., Lusaka, Zambia; Ministry of Health, Ndeke House, Haile Selassie Avenue, PO Box 30205, Lusaka, Zambia; Zambart, Health Economics Unit, Ridgeway, Zambia; ICARS, International Centre for Antimicrobial Resistance Solutions, Ørestads Boulevard 5, Copenhagen 2300, Denmark

## Abstract

Despite the escalating burden of antimicrobial resistance (AMR), the global response has not sufficiently matched the scale and scope of the issue, especially in low- and middle-income countries (LMICs). While many countries have adopted national action plans to combat AMR, their implementation has lagged due to resource constraints, dysfunctional multisectoral coordination mechanisms and, importantly, an under-recognized lack of technical capacity to adapt evidence-based AMR mitigation interventions to local contexts. AMR interventions should be tailored, context-specific, cost-effective and sustainable. The implementation and subsequent scale-up of these interventions require multidisciplinary intervention-implementation research (IIR). IIR involves both quantitative and qualitative approaches, occurs across a three-phase continuum (proof of concept, proof of implementation and informing scale-up), and across four context domains (inner setting, outer setting, stakeholders and the implementation process). We describe the theoretical underpinnings of implementation research (IR), its various components, and how to construct different IR strategies to facilitate sustainable uptake of AMR interventions. Additionally, we provide real-world examples of AMR strategies and interventions to demonstrate these principles in practice. IR provides a practical framework to implement evidence-based and sustainable AMR mitigation interventions.

## Antimicrobial resistance: the silent pandemic

Antimicrobial resistance (AMR) is ranked among the top 10 threats to global health by the WHO.^1,2^ It is potentially the greatest public health threat of our time, surpassing COVID-19 because of its continuing and progressive nature with extensive adverse effects on the health of humans, animals, crops and the environment.^3–7^ From a human health perspective, a world without effective antimicrobial medicines would severely compromise healthcare as we know it, limiting our ability to perform major surgeries, conduct organ transplantations, treat premature babies and administer cancer chemotherapies.^8^ AMR further affects animal health and welfare, food security and food safety. In 2019 alone, 1.27 million deaths were estimated to be directly attributable to AMR globally.^9^ The Independent O’Neill Review estimates an annual mortality rate of up to 10 million by 2050 due to AMR, with up to 9 million deaths disproportionately occurring in low- and middle-income countries (LMICs) unless immediate and effective action is taken.^10^ Furthermore, the World Bank estimates that AMR could result in an additional 28 million people living in severe poverty, a 7.5% decline in global livestock production, a 3.8% reduction in global exports and 1 trillion USD in additional healthcare costs by 2050.^11^ Yet the negative impact of AMR has not engendered adequate and sustainable action, politically or otherwise, especially in LMICs, as AMR is somewhat intangible and frequently described as a silent pandemic, despite the high burden.^10^ The COVID-19 crisis provides a foretaste of what AMR can mean to the world without appropriate interventions and the human capital to implement them, making pandemic preparedness for AMR imperative.^12–14^

## National action plans on AMR

In 2015, collaboration between the Tripartite consisting of the WHO, the Food and Agriculture Organization (FAO) of the United Nations, and the World Organisation for Animal Health (OIE) resulted in the Global Action Plan (GAP) on AMR.^15^ The World Health Assembly Resolution 68.7 (WHA68.7) urged member states to have in place national action plans (NAPs) on AMR aligned to the GAP by the 70th World Health Assembly in May 2017,^16^ and in September 2016, the United Nations General Assembly signed the Political Declaration on Antimicrobial Resistance (AMR) that endorsed WHA68.7.^17,18^

In its April 2019 final report, the UN Inter-Agency Coordination Group (IACG) on AMR strongly recommended that accelerated implementation of NAPs ‘must be at the heart of the global response to AMR’.^19^ The report, however, acknowledged that significant challenges remain in the implementation of the NAPs, with few countries having set up functional multisectoral coordination mechanisms and even fewer countries financing their NAPs.^19^

According to the latest Tripartite AMR country self-assessment survey (TrACSS), 149 countries have developed NAPs on AMR.^20^ However, the translation of policy to action has not sufficiently matched the scale and scope of the issue. Implementation of NAPs is particularly challenging in LMICs that require substantial development assistance and the whole-of-government ownership to implement their NAPs at scale. Long-term ownership and sustainability of these investments at national level when development funding ceases is a further challenge. LMICs lag behind high-income countries (HICs) in all indicators on the implementation and financing of the NAPs as evident from the country self-assessment reports based on the Tripartite monitoring tool.^21^

One crucial step to start implementation of NAPs is for countries to develop, test and/or adapt interventions to mitigate AMR. Although there is a growing body of evidence on effective AMR mitigation interventions,^22–24^ this evidence has been largely developed in high-resource settings and HICs and cannot be directly translated to LMICs or often even between HICs. Mitigating AMR in LMICs requires tailored, context-specific, cost-effective and sustainable interventions.^25^ This paper proposes the use of implementation research (IR) to provide proof of concept of AMR mitigation interventions in local contexts with the aim of sustainable scale-up.

## What is IR?

IR is defined as ‘the scientific inquiry into questions concerning implementation—the act of carrying an intention into effect, which in health research can be policies, programmes, or individual practices (collectively called interventions)’.^23^ When adapted for AMR, IR may involve the quantitative and/or qualitative scientific validation of processes that will facilitate the systematic and sustainable uptake of evidence-based AMR interventions into routine practice. The ultimate goal of IR in AMR is to improve the capacities of human, animal, agricultural and environmental health systems to mitigate AMR individually and collectively in a coordinated One Health approach.^26,27^ The achievement of this goal requires human capital development in IR in addition to financial resources.

**Figure 1.**
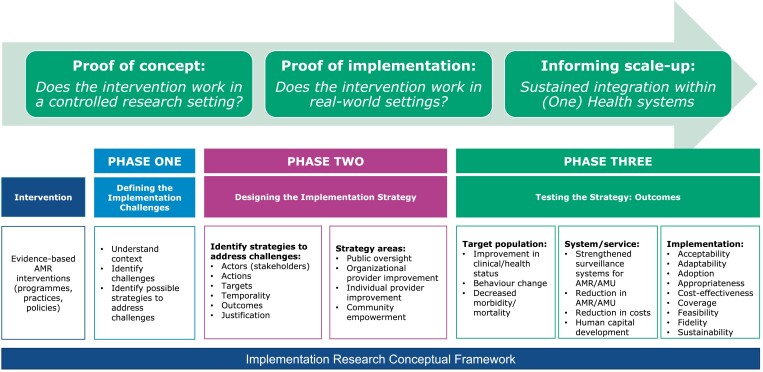
IR conceptual framework.

IR occurs across a three-phase continuum (Figure 1): proof of concept, proof of implementation and informing scale-up; and four context domains: inner setting, outer setting, stakeholders involved and the implementation process, all of which influence the implementation of intervention(s).^27,28^ The IR strategy involves three distinct steps: defining the IR challenge, designing the implementation strategy and testing the implementation strategy. The strategy defines the actors, actions, targets and temporality, and determines outcomes at three levels: target population level outcomes, system/service level outcomes and implementation outcomes.^29,30^ Each of these components is described below with illustrative examples.

## IR continuum

Phase one of the continuum provides proof of concept, i.e. does the intervention work in a controlled research setting? Proof of concept is usually associated with basic science, product development, Phase I and II clinical trials, or qualitative studies such as perceptions of illness or quality of health/veterinary services. Research is undertaken in a fully controlled setting such as a laboratory or amongst a defined population where implementation strategies and variables are not relevant. Phase two explores proof of implementation, i.e. does the intervention work in real-world settings in different contexts? Proof of implementation determines the effectiveness of an intervention using effectiveness-implementation trials, observational studies, or participatory research. Research is undertaken in different real-world settings and populations and is partially controlled. Here, implementation strategies and variables are important research components since interventions may work in one setting but not in others. Phase three focuses on informing scale-up, i.e. sustained integration within systems. Scale-up is generally informed by mixed methods, quasi-experimental studies, or observational studies to determine the enablers of and barriers to sustained scale-up. Research is undertaken in a real-world setting and population and implementation strategies and variables are the main or only research focus.^30^

The discovery and path to the clinical use of penicillin illustrates this continuum. In 1928, Alexander Fleming, following his return from vacation, serendipitously observed a zone on an agar plate around an invading fungus without any staphylococcal growth.^31^ Fleming isolated the mould and identified it as belonging to the *Penicillium* genus, naming its active agent penicillin. While he published his findings in 1929,^32^ he was unable to further purify the compound for therapeutic use.^31^ In the late 1930s, Ernst Chain, Howard Florey and Norman Heatley of the University of Oxford successfully isolated, purified and produced penicillin based on Fleming’s original work.^33^ They then proceeded to test the compound on mice infected with *Streptococcus* isolates and found that the compound had a bactericidal effect, publishing their findings in 1940.^31,34^ This process reflects phase one of the IR continuum, providing a proof of concept for penicillin in a controlled setting, similar to Phase I and II clinical trials known today. In 1941, a local policeman with a severe infection was one of the first human subjects to receive treatment with penicillin.^35^ While his condition initially improved, it worsened as the limited supply of penicillin ran out. Following this initial demonstration of effect, other patients were subsequently successfully treated with the compound,^36^ demonstrating the effectiveness of the compound in a clinical setting and representing proof of implementation for penicillin. However, the mass production and use of penicillin remained a challenge. This required scale-up, representing phase three of the IR continuum, for which Florey and Heatley travelled to the USA.^31^ Together with scientists from the US Department of Agriculture, and later the US government, production methods were quickly improved, expanding penicillin supplies exponentially. By September 1943, the stock of penicillin was sufficient to cover the needs of the Allied Armed Forces.^37^ The development of penicillin is thus a prime example of the three main phases of the IR continuum: proof of concept, proof of implementation and informing scale-up.

## Context domains

**Figure 2.**
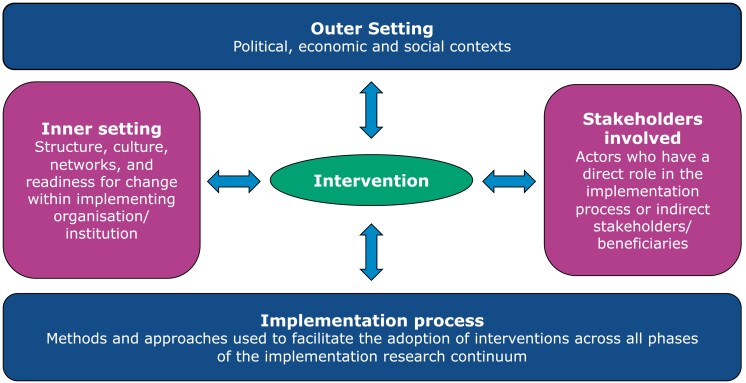
Context domains of IR (adapted from the WHO Implementation Research Toolkit).

Two key constructs of IR are context and stakeholder inclusion to facilitate implementation and sustainable integration of successful interventions into existing systems. Interventions are implemented within and between four context domains: the outer setting, the inner setting, the stakeholders involved and the implementation process (Figure 2). The economic, political and social contexts in which an intervention is carried out constitute the outer setting, which usually cannot be controlled by the implementing organization/institution/system. The structure, culture, networks and readiness for change within the implementing organization/institution/system is the inner setting. All stakeholders involved in any part of the IR continuum constitute a critical context as their knowledge, attitudes and perceptions to the intervention and its implementation will influence its success and impact. The implementation process is the core context domain and incorporates all the strategies used in facilitating the adaptation and adoption of the intervention across all phases of the continuum, including those explicitly planned as well as the unintended ones that emerge during implementation. The inter-related context domains highlight the complexity of real-life environments requiring an understanding of how to navigate social, economic, political, system and organizational contexts with a diversity of stakeholders at multiple levels.^38^

The second key construct relates to the deliberate inclusion of all stakeholders that have a direct or indirect role in the implementation process and/or are potential direct/indirect beneficiaries from project inception. They include but are not limited to government ministries, policymakers, administrators, human, animal and environmental health practitioners/providers, patients, farmers and civil society,^39^ requiring a concurrent top-down and bottom-up approach to IR from inception to scale-up.^40^

Early engagement and collaboration with stakeholders at all levels is key to implementing and integrating interventions into existing systems, ensuring that contextual considerations are integrated from inception. Stakeholders and researchers co-develop an in-depth understanding of local challenges and bottlenecks, jointly identify relevant research questions and frame interventions within local contexts and available resources, ensuring ownership and commitment to scale-up.^39^

A project developed by AMR researchers from a Tanzanian university in partnership with the Tanzanian Ministry of Livestock and Fisheries illustrates the importance of context and stakeholder engagement. Given the increasing demand for poultry products in Tanzania, the intensive poultry industry is experiencing steady growth.^41,42^ Farmers rely on prophylactic and metaphylactic antimicrobials to maintain flock health and increase productivity in the absence of adequate and effective biosecurity and vaccination practices.^43,44^ The most frequently used antimicrobials are sulphonamides and tetracyclines, with the consequent risk of AMR.^45^ A project focusing on disease prevention using poultry vaccination and biosecurity interventions (Table S1, available as Supplementary data at *JAC-AMR* Online) was developed with due consideration of context and stakeholder engagement (Table 1).

**Table 1.** Context domains and stakeholders of the Tanzanian intensive poultry industry

Context	Description
Outer setting	Policy context: Government priorities for AMR mitigation are set by the Tanzanian National Action Plan on AMR, which outlined 10 action packages to combat AMR.^46^ While progress has been made, there remains an important implementation gap in action packages that address the root cause of AMR in agriculture and poultry production. National policies, regulations and guidelines that may influence the poultry production industry include, but are not limited to, the National One Health Strategic Plan,^47,48^ Tanzania Livestock Master Plan (2017/2018–2021/2022),^49^ National Livestock Research Agenda 2020–2025,^50^ National Livestock Policy (2006),^51^ The Animal Diseases (Hatcheries and Breeding flock farms) Regulations (2019)^52^ and The Grazing-Land and Animal Feed Resources Act.^53^ Economic context: The growing demand for poultry meat and eggs as a healthier and cheaper alternative to other meat products has led to growing economic opportunities for the poultry industry.^41,42^ Policy interventions in Tanzania are also expected to stimulate further growth in the private sector.^49^
Inner setting	The inner setting is intensive poultry production farms, who are willing to explore strategies to reduce AMU by implementing vaccination and other biosecurity programmes.
Stakeholders	Individual actors: Policymakers Poultry farmers and other farming personnel Veterinary personnel Researchers Organizational actors: Ministry of Livestock and Fisheries Local government authorities National Poultry Association Poultry Breeders Association University Vaccine providers
Implementation process	Conduct cross-sectional qualitative (key-informant interviews) and quantitative surveys (questionnaires) to understand knowledge, practices, behaviours and skills to tailor interventions to local realities. Conduct a cluster RCT (cRCT) to test the implementation of the intervention package (vaccinations and other biosecurity programmes). Build a business model for implementing the interventions. Conduct local capacity building of farmers and researchers. Disseminate research findings with other community stakeholders in popular media, peer-reviewed journals and stakeholder meetings.

## IR strategy

Step one of the IR strategy involves defining the IR challenges with relevant stakeholders in the specific practice/systems context in which an evidence-based intervention is to be implemented, as described under ‘Context Domains’ above. Steps two and three, respectively, involve designing and testing the strategy (Figure 1).

The strategy design identifies actors (stakeholders) both top-down and bottom-up, actions (steps or processes to sustainably implement the intervention), targets (beneficiaries and/or implementers of the intervention) and temporality (chronology of the implementation process). The design also includes the identification of enablers of and barriers to implementation.^54^ Broad strategy areas include but are not limited to public oversight, organizational provider improvement, individual provider improvement, and household and community empowerment.^26^

Common research methods are pragmatic trials, effectiveness-implementation hybrid trials, quality improvement studies, participatory action research and mixed methods. Randomized controlled trials (RCTs) typically evaluate the efficacy of an intervention in an ‘ideal’ or controlled setting with narrowly defined inclusion and exclusion criteria and focus on clinical outcomes. Pragmatic or practical trials are RCTs that evaluate the effectiveness of an intervention in the real-world setting with all the relevant stakeholders. Effectiveness-implementation hybrid trials assess the effectiveness of the intervention and implementation strategy in tandem. There are three hybrid research designs: type 1 assesses the effects of an intervention on relevant target or system outcomes while observing and gathering information on implementation in terms of the feasibility and acceptability of the implementation approach through qualitative, process-oriented or mixed-methods study designs; type 2 involves testing of health interventions and implementation strategies equally; and type 3 primarily evaluates the implementation strategy while observing and gathering information on the impact of the intervention on the relevant target or system outcomes.

Quality improvement studies usually take the form of the structured and iterative plan-do-study-act cycle that develops (plan) and implements (do) a plan, as well as analyses and interprets the results (study) to inform next steps (act). Participatory action research (PAR) ensures that implementation occurs with and by the relevant stakeholders at all levels such that stakeholders have power and control over the implementation process. PAR is usually qualitative in nature, but quantitative and mixed-methods techniques are increasingly being used. Mixed methods involve both qualitative and quantitative methods of data collection and analysis in the same study. Mixed methods are particularly suitable for IR because they provide practical ways to understand several perspectives, diverse causal pathways and multiple types of outcomes.^26^

The strategy is tested against predetermined implementation outcomes such as one or more of acceptability, adaptability, adoption, appropriateness, costs, coverage, feasibility, fidelity (the extent to which an intervention was implemented as described in the IR protocol) and sustainability.^26^ The IR strategy may additionally be tested against target-level and system/service outcomes. The former may include improvements in health status, behaviour change, a decrease in morbidity or improvement in knowledge, attitudes and practices.^55^ The latter may include strengthened and/or integrated One Health surveillance systems for AMR and antimicrobial use (AMU), a reduction in AMR, AMU and hospital-acquired infections (HAIs), improved biosecurity, hygiene and sanitation, and human capital development in AMR mitigation (Figure 1). Measuring implementation outcomes improves the understanding of implementation processes, allows comparison of the effectiveness of different implementation strategies and differentiates between intervention failure and implementation failure.^29,56^ Examples of implementation strategies are illustrated in Table 2 with additional examples in Tables S1 and S2.

**Table 2.** Illustrative examples of implementation strategies, adapted from project proposals supported by the International Centre for Antimicrobial Resistance Solutions

IR strategy concepts	Human health	Animal health	Environmental health
Project title	Facilitating appropriate antibiotic use in respiratory tract infections in children in Kyrgyzstan	Reducing post-weaning diarrhoea and antimicrobial use through improved provision of colostrum and use of vaccines in weaning pigs in Colombia	Mitigating the spread of antimicrobial residues and resistant microbes through the treatment of manure
Defining the AMR challenge			
	Inappropriate antibiotic use has been documented among children presenting to Kyrgyz primary healthcare centres.^57^ The aim of the project is to reduce unnecessary prescription of antibiotics in children with respiratory tract infections (RTIs).	The pig industry is a major user of antimicrobials, particularly to control weaning diarrhoea due to the weak immune system of piglets. This drives inappropriate use of antimicrobials. The aim of the project is to evaluate the effect of two interventions alone and in combination, i.e. improved uptake of colostrum and vaccines in reducing the incidence of diarrhoea and the need to use antimicrobials.	In Tanzania, most antimicrobials in poultry are administered orally, of which 70%–90% are excreted in manure.^58,59^ This may lead to environmental contamination with antimicrobial residues and antimicrobial-resistant pathogens and genes, which are food safety risks. The poultry industry is rapidly expanding in Tanzania, and in parallel, poultry manure is a desirable fertilizer (e.g. for short-cycle crops). The aim of the project is to optimize manure processing solutions to curb environmental contamination while increasing the demand for processed manure by crop/fish farmers.
Designing the strategy			
* *Strategy area	Individual provider improvement (clinicians, caregivers) Supporting multiple stakeholders engaged in improving health (clinicians, laboratory staff, caregivers) Enhancing the performance of implementing and provider organizations (primary healthcare centres)	Individual provider improvement Supporting multiple stakeholders engaged in improving (One) Health Enhancing the performance of implementing and provider organizations	Individual provider improvement (farmers, manure composters) Supporting multiple stakeholders engaged in improving (One) Health (poultry farmers, crop/fish farmers, manure composters, policymakers)
* *Research methodology	RCT Observational study Qualitative study Situational analysis of knowledge, attitude and practices (KAP) of prescribers Economic analysis; cost savings, cost–benefit, and cost-effectiveness assessment	RCT Qualitative study Economic analysis	Situational analysis of KAP and behaviours of farmers, manure composters, and the regulatory context cRCT Cost–benefit analysis and willingness to pay assessment to develop a business case
* *Actors	Individual actors: Clinicians Caregivers Laboratory staff Researchers Organizational actors: Ministry of Health University	Individual actors: Policymakers Veterinarians Farm workers Pig producers Organizational actors: Ministry of Agriculture Agricultural Institute Private sector pig producers	Individual actors: Policymakers Poultry farmers Manure composters Crop/fish farmers Researchers Organizational actors: Ministry of Agriculture Local government authorities Small business/potential entrepreneurs Agricultural Civil Society Organizations University
* *Actions	Conduct an RCT to test the effectiveness of using a C-reactive protein (CRP) point-of-care test (POCT) as a decision support diagnostic tool for local healthcare workers to decrease inappropriate antibiotic use. Conduct an economic study to assess the cost-effectiveness of the intervention. Conduct a mixed-methods cross-sectional study to assess KAP among prescribers pre-and post-intervention. Conduct an observational study to identify pathogens causing RTIs in children in the study areas. Conduct qualitative interviews to ascertain enablers and barriers to implementation as well as other implementation research outcomes. Disseminate research findings with other stakeholders in popular media, peer-reviewed journals and stakeholder meetings.	Conduct an RCT to evaluate the effect of different interventions (colostrum provision, vaccination of sows etc.) in comparison with standard herd health management practices. Conduct an economic study to assess the cost–benefit of different interventions. Conduct a qualitative study to identify behavioural and contextual factors facilitating and limiting the implementation and potential scale-up of the proposed interventions. Disseminate research findings with other stakeholders in popular media, peer-reviewed journals and stakeholder meetings.	Conduct a mixed-methods cross-sectional study to identify behaviours, knowledge and skills on poultry manure use and associated risks from manure and AMR. Assess regulatory/legislative barriers and opportunities for adopting optimized manure processing technologies. Conduct a cRCT to evaluate the effect of different composting strategies. Conduct an economic analysis to develop a business case for entrepreneurs and small businesses to engage in composting. Conduct training for capacity building of the local workforce. Disseminate research findings with other stakeholders in popular media, peer-reviewed journals and stakeholder meetings.
* *Targets	Clinicians (improve prescribing practice) Patients (receive appropriate antimicrobial treatment) Researchers (capacity building)	Piglets (reduce post-weaning diarrhoea) Private sector pork producers (reduce the use of antimicrobials) Policymakers (strengthen legislation) Researchers (capacity building)	Farmers (use manure subjected to composting) Manure composters and small businesses (implement composting practices) Agricultural associations (facilitate the demand for composting manure) Policymakers (strengthen legislation) Researchers (capacity building)
Testing the strategy			
* *IR outcomes	Acceptability Adoption Appropriateness Cost-effectiveness Feasibility	Acceptability Adoption Cost-effectiveness Feasibility	Acceptability Adoption Appropriateness Cost-effectiveness Feasibility
* *System-level outcomes	Optimize antibiotic prescription in children with RTIs. Improve the capacity of laboratories to conduct microbiological culture and susceptibility for RTIs. Map the common causative RTI pathogens, their antibiotic susceptibility profiles and clinical presentation to inform treatment options. Strengthen AMR research capacity at individual and organizational levels.	Reduce the use of antimicrobials in intensive pig production. Strengthen AMR research capacity at individual and organizational levels.	Increase demand for processed manure by crop/fish farmers. Build a business case for value-added poultry manure from farm to end-users. Strengthen existing regulatory frameworks that support increased uptake of processed and safe fertilizer from poultry manure. Strengthen AMR research capacity at individual and organizational levels.
* *Target-level outcomes	Improve the knowledge, attitudes and prescribing practices of different healthcare workers for RTIs in children. Raise the level of knowledge and training of medical workers on the role of diagnostics and the rational use of antibiotics in clinical practice. Reduce inappropriate use of antibiotics in children with RTIs.	Reduce the use of antimicrobials in pig production. Strengthen AMR research capacity at individual and organizational levels.	Enhance the KAP of commercial poultry manure farmers/processors on current use of poultry manure and associated potential food safety risks. Improve the behaviour and skills of crop/fish farmers towards the use of poultry manure. Optimize manure composting technology for the Tanzanian context.

These examples illustrate the different characteristics of IR research that require researchers and implementers to have a strong understanding of contextual realities at the user and policy levels, highlighting the importance of the outer setting, inner setting, the stakeholders and the actual implementation process, all of which will have an impact on the (un)successful implementation of evidence-based AMR interventions. The examples also highlight that no single aspect of the context exists in isolation, and that successful scale-up requires a bottom-up and top-down approach that is grounded in local realities.

## Conclusions

The implementation of AMR mitigation interventions is undoubtedly affected by resource constraints—particularly in LMICs. Implementation is also constrained by the under-recognized lack of technical capacity to adapt and adopt evidence-based AMR mitigation policies, programmes and practices to local country contexts. Incentivizing stakeholders to implement and sustainably integrate evidence-based AMR interventions may be advanced by ‘small tests of change’ in the form of pilot projects where the implementing organization/system has a preview of the outcomes, specifically feasibility and cost-effectiveness, before organization/system-wide implementation or scale-up. Investments in human capital development in IR is critical to ensuring that projects can be adapted to changes in local contexts and sustained in the long term.

IR thus provides a practical framework to address AMR across unique settings. IR highlights the interface between theory and practice, addressing the ‘know-do’ gap. It is context-specific, demand-driven and works at a multidisciplinary level. IR is undertaken in the real world in real time, inclusive of all stakeholders—using research designs and methodologies that are fit for purpose and include both process and outcome indicators.^60^

## Supplementary Material

dlad031_Supplementary_Data

## Supplementary data

Tables S1 and S2 are available as Supplementary data at *JAC-AMR* Online.
