# Supplementary material for: Mitigating antimicrobial resistance (AMR) using implementation research: a development funder’s approach
Source: JAC Antimicrob Resist. 2023 Mar 27;5(2):dlad031. doi: 10.1093/jacamr/dlad031 (PMC10041058; doi:10.1093/jacamr/dlad031)
Supplement: dlad031_Supplementary_Data [file dlad031_supplementary_data.docx]

**Supplementary File**

**Supplementary Table S1:** Further examples of implementation strategies for Animal Health

| **IR Strategy Concepts** | **Animal Health in Tanzania** | **Animal Health in Zambia** | **Animal Health in Georgia** | **Animal Health in Vietnam** |
| --- | --- | --- | --- | --- |
| **Project Title** | - Reducing antimicrobial use through promotion and implementation of vaccination and biosecurity programs in intensive poultry production farms | - Combating Antimicrobial Resistance and Antimicrobial Residues in the Zambian Poultry Sector | - Reducing the use of antimicrobial growth promoters (AGPs) on Poultry Farms in Georgia | - Reduction of colistin use in Vietnamese pig production |
| **Defining the challenge** | | | | |
| **Defining the AMR challenge** | - The increased demand for eggs and broiler meat requires intensification of poultry production, a practice that increases the risk of poultry diseases if proper preventive and control measures are not instituted - The aim of the project is to reduce antimicrobial use in commercial poultry production through the promotion and implementation of vaccination and biosecurity programs that are feasible, cost-effective, and sustainable for the Tanzanian setting. | - Growing consumer demand for poultry products in Zambia has contributed to non-prudent antimicrobial use on farms to prevent and treat poultry diseases. - Other drivers of antimicrobial use include limited knowledge of other disease prevention strategies, inadequate resources to guide alternative approaches amongst farmers and veterinary personnel, and easy to access to antibiotics through informal providers. - The aim of this project is to optimise antimicrobial prescribing, provision, and use among the veterinary personnel, agro-veterinary shop sellers, and farmers respectively. | - Georgia lacks a nationwide surveillance system for monitoring AMR and AMU in the animal sector. There is no direct ban preventing the use of antimicrobials as growth promoters in feed, nor the use of welcome antibiotics for day-old chicks upon arrival at broiler farms. - The aim of the project is to demonstrate that eliminating the use of antibiotics as growth promoters and welcome antibiotics on broiler farms in Georgia does not compromise animal welfare, productivity, or income. | - Colistin is currently used in Vietnam for prevention and control of neonatal and post-weaning diarrhoea (PWD) associated with *Escherichia coli.* - The aim of the project is to support cost-effective and sustainable pig production with reduced use of colistin and other antibiotics. |
| **Defining the Strategy** | | | | |
| - Strategy Area | - Individual provider improvement (veterinarians) - Organizational provider improvement (private vaccine providers) - Enhancing the performance of implementing and provider organizations (vaccine supply chain) | - Individual provider improvement (veterinary personnel, agro-veterinary shop sellers, farmers) - Supporting multiple stakeholders engaged in improving (One) Health (as above) - Enhancing the performance of implementing and provider organizations (intensive poultry production farms) | - Individual provider improvement (farmers, veterinarians) - Enhancing the performance of implementing and provider organizations (intensive poultry production farms) - Supporting multiple stakeholders engaged in improving (One) Health (as above) | - Individual provider improvement (pig farmers) - Organizational provider improvement (intensive pig producers) |
| - Research methodology | - Situational analysis - Cluster randomized controlled trial (cRCT) - Economic analysis | - Situational analysis - Pre/post intervention study - Economic analysis | - Qualitative study - Equivalence trial - Economic analysis | - Situational analysis - Intervention trial - Policy analysis - Economic analysis |
| - Actors | Individual Actors:   - Policymakers - Veterinarians - Poultry farmers - Researchers   Organizational Actors:   - Ministry of Livestock and Fisheries - Private vaccine provider - University | Individual Actors:   - Veterinary personnel - Farmers - Agro-vet shop sellers   Organizational Actors:   - Ministry of Agriculture - Veterinary Research Institute - University - Veterinary Association - National Medicines Regulatory Authority | Individual Actors:  - Veterinarians - Farmers  Organizational Actors:Ministries of Agriculture and the EnvironmentResearch InstituteState LaboratoryPrivate providers | Individual Actors:   - Policymakers - Pig farmers - Veterinarians - Researchers   Organizational Actors:   - Ministry of Agriculture  Intensive pig producers  - University |
| - Actions | - Conduct a cross-sectional qualitative (key-informant interviews) and quantitative surveys (questionnaires) to understand knowledge, practices, behaviours, and skills to tailor interventions to local realities. - Conduct a cluster randomized controlled trial (cRCT) to test the implementation of the intervention package (vaccinations and other biosecurity programmes). - Build a business model for implementing the interventions. - Conduct local capacity building of farmers and researchers. - Disseminate research findings with other stakeholders in popular media, peer reviewed journals, and stakeholder meetings. | - Conduct a baseline mixed methods situational analysis to assess current knowledge, behaviours, and practices. - Develop tailored training interventions (e.g. workshops, farmer field schools) to facilitate behaviour-change amongst veterinary personnel, farmers and agro-vet sellers. - Develop and disseminate comprehensive treatment guidelines for veterinarians. - Assess changes in residues in poultry meat, eggs, and feed. - Conduct economic analyses to determine the feasibility and sustainability for scaling up the project. - Disseminate research findings with other stakeholders in popular media, peer reviewed journals, and stakeholder meetings. | - Develop a questionnaire to collect information on flow of inputs into the farm system, including source of feed, and whether it is imported or locally produced feed to inform the equivalence trial. - Conduct an equivalence trial to determine whether raising poultry without AGPs and welcome antibiotics results in similar weight and mortality outcomes compared to traditional rearing methods. - Conduct a qualitative assessment to map barriers and facilitators for reducing/eliminating AGPs and welcome antibiotics to inform scale-up strategies. - Conduct an economic evaluation to assess the cost-effectiveness and potential cost savings of different scenarios to phase out AGPs and welcome antibiotic use. - Disseminate research findings with other stakeholders in popular media, peer reviewed journals, and stakeholder meetings. | - Determine sources and quantify import, export, and on-farm use of colistin. - Conduct on-farm vaccination and antibiotic alternative intervention trials to document their sustainability and cost-effectiveness to control piglet diarrhoea. - Undertake an assessment of animal health and economic risks to pig farmers. - Identify required changes in current legislation to support the reduced use of colistin. - Disseminate research findings with other stakeholders in popular media, peer reviewed journals, and stakeholder meetings. |
| - Targets | - Policymakers (strengthen legislation) - Poultry farmers (improved biosecurity measures) - Veterinarians (improved knowledge and practice) - Researchers (capacity building) | - Veterinary personnel, farmers, and agro-vet shop sellers (improved prescribing, use, and provision of antimicrobials) - Researchers (capacity building) | - Policymakers - (strengthen legislation) - Veterinarians (optimize antimicrobial prescribing) - Farmers (optimize antimicrobial use) | - Policymakers (strengthen and enforce legislation) - Pig farmers (increased vaccine and decreased antibiotic use) - Researchers (capacity building) |
| **Testing the Strategy** | | | | |
| - IR Outcomes | - Acceptability - Adoption - Cost-effectiveness - Feasibility - Sustainability | - Acceptability - Adoption - Appropriateness - Cost-effectiveness - Coverage - Feasibility - Sustainability | - Acceptability - Appropriateness - Cost-effectiveness - Feasibility - Scalability - Sustainability | - Acceptability - Adoption - Cost-effectiveness - Feasibility - Sustainability |
| - System Level Outcomes | - Improve existing policy and regulatory frameworks that govern the use of vaccines and   biosecurity measures in commercial poultry production.   - Increase the effectiveness of defined vaccination and other biosecurity programmes to reduce the occurrence of diseases and antimicrobial use. - Establish a sustainable business model to scale-up interventions. | - Reduce AMU and residues in poultry meat and eggs to improve food safety. | - Provide relevant decision-makers with the information needed to introduce legislation to ban AGPs and welcome antibiotics. | - Increase the understanding of effective intervention measures across different farm settings to reduce colistin use and control piglet diarrhoea while maintaining efficient production and pig health management. - Increase knowledge for decision-making to control piglet diarrhea with reduced antibiotic use. |
| - Target Level Outcomes | - Increase knowledge, attitudes, and practices of stakeholders in the value chain on appropriate   vaccination and other biosecurity programmes in commercial poultry production.   - Improve capacity through training and research knowledge transfer. | - Improve antimicrobial knowledge and use practices amongst veterinary personnel, farmers, and agro-vet medicine sellers. - Increase prudent prescription use of antimicrobials among veterinary personnel through formulated treatment guidelines. | - Increase farmers’ and veterinarians’ knowledge and ability to raise poultry without AGPs and welcome antibiotics. - Improve farmers’ and veterinarians’ knowledge of the economic benefits of phasing out use of AGPs and welcome antibiotics. | - Increase knowledge and capacity within government to estimate import, export, and antibiotic use. - Improve the control of piglet diarrhoea using vaccination and non-colistin antibiotics. |

**Supplementary Table S2:** Further examples of implementation strategies for Human Health

| **IR Strategy Concepts** | **Human Health in Zambia** | **Human Health in Georgia** |
| --- | --- | --- |
| **Project Title** | - Optimisation of antimicrobial use in BSIs and UTIs in Various Health Sector Settings in Zambia | - Improving the quality of surgical antibiotic prophylaxis [SAP] in a selection of Georgian hospitals – A pilot AMS intervention |
| **Defining the challenge** | | |
| **Defining the AMR challenge** | - Urinary tract infections (UTIs) and bloodstream infections (BSIs) have been recognized as clinical syndromes associated with inappropriate and extensive use of antibiotics which contributes to antibiotic resistance (ABR). - The aim of the project is to optimize the use of antibiotics in UTIs and BSIs by introducing an antimicrobial stewardship (AMS) programme across the continuum of care. | - In Georgia, there is inappropriate use of antibiotics for surgical prophylaxis, as well as extensive and prolonged use of broad-spectrum antibiotics. - The project aims to improve the quality of the use of antibiotics for surgical prophylaxis in Georgian hospitals by implementing a multimodal intervention based on the WHO antimicrobial stewardship tool kit. |
| **Defining the Strategy** | | |
| - Strategy Area | - Individual provider improvement (clinicians, pharmacists, nurses, clinical officers) - Organizational provider improvement (primary healthcare centres, referral hospitals) - Enhancing the performance of implementing and provider organizations (as above) | - Individual provider improvement (clinicians) - Enhancing the performance of implementing and provider organizations (hospitals) |
| - Research methodology | - Observational study - Qualitative study - Economic analysis | - Quality improvement study - Qualitative study - Economic analysis |
| - Actors | Individual Actors:   - Policymakers - Regulatory Agency - Hospital managers - Clinicians - Pharmacists - Nurses - Clinical Officers - Patients - Researchers   Organizational Actors:   - Ministry of Health - Global partners working in AMS (ReACT Africa) - University | Individual Actors:   - Policymakers - Public Health practitioners - Hospital managers - Clinicians - Pharmacists - Patients - Researchers   Organizational Actors:   - Ministry of Health - Professional associations - Private research organization - University |
| - Actions | - Undertake a situational analysis to obtain baseline data on AMU and compliance to guidelines. - Conduct capacity building and training of the multidisciplinary AMS team to facilitate the implementation of an effective and sustainable AMS programme using the Plan–Do–Study–Act-Adjust framework (PDSAA). - Conduct an economic evaluation. | - Establish active surveillance for surgical site infections (SSIs). - Conduct Point Prevalence Surveys (PPS) and prescription audits to assess and reflect on compliance to guidelines. - Conduct qualitative interviews with all stakeholders and undertake cost-benefit analyses to explore the barriers and enablers to implementing and scaling up the intervention. |
| - Targets | - Policymakers (strengthen legislation) - Hospital managers (adopt AMS programmes) - Prescribers (improve prescribing practice) - Patients (receive appropriate antimicrobial treatment) | - Clinicians (improve prescribing practice) - Patients (receive appropriate antimicrobial treatment) |
| **Testing the Strategy** | | |
| - IR Outcomes | - Adoption - Appropriateness - Cost-effectiveness - Feasibility - Sustainability | - Adoption - Appropriateness - Cost-effectiveness - Feasibility - Sustainability |
| - System Level Outcomes | - Strengthen health systems in AMS - Optimize AMU - Strengthen AMR and AMS research capacity at individual and organizational levels. | - Strengthen surveillance of SSIs - Optimize antibiotic use for SAP - Strengthen AMR and AMS research capacity at individual and organizational levels. |
| - Target Level Outcomes | - Improve knowledge, attitudes, and practices (KAP) of prescribers. - Develop skills of prescribers in AMS along the continuum of care. | - Improve awareness, knowledge, and clinical practice on SAP and SSIs amongst clinicians. |
